# *Synechocystis* sp. PCC 6803 overexpressing genes involved in CBB cycle and free fatty acid cycling enhances the significant levels of intracellular lipids and secreted free fatty acids

**DOI:** 10.1038/s41598-020-61100-4

**Published:** 2020-03-11

**Authors:** Kamonchanock Eungrasamee, Aran Incharoensakdi, Peter Lindblad, Saowarath Jantaro

**Affiliations:** 1 Laboratory of Cyanobacterial Biotechnology, Department of Biochemistry, Faculty of Science, Chulalongkorn University, Bangkok, 10330 Thailand; 2 Microbial Chemistry, Department of Chemistry – Ångström, Uppsala University, Box 523, SE-75120 Uppsala, Sweden

**Keywords:** Lipids, Metabolic engineering

## Abstract

The integrative aspect on carbon fixation and lipid production is firstly implemented in cyanobacterium *Synechocystis* sp. PCC 6803 using metabolic engineering approach. Genes related to Calvin–Benson–Bassham (CBB) cycle including *rbcLXS* and *glpD* and free fatty acid recycling including *aas* encoding acyl-ACP synthetase were practically manipulated in single, double and triple overexpressions via single homologous recombination. The significantly increased growth rate and intracellular pigment contents were evident in *glpD*-overexpressing (OG) strain among all strains studied under normal growth condition. The triple *aas_glpD_rbcLXS*-overexpressing (OAGR) strain notably gave the highest contents of both intracellular lipids and extracellular free fatty acids (FFAs) of about 35.9 and 9.6% w/DCW, respectively, when compared to other strains at day 5 of cultivation. However, the highest intracellular lipid titer and production rate were observed in OA strain at day 5 (228.7 mg/L and 45.7 mg/L/day, respectively) and OG strain at day 10 (358.3 mg/L and 35.8 mg/L/day, respectively) due to their higher growth. For fatty acid (FA) compositions, the main saturated fatty acid of palmitic acid (C16:0) was dominantly found in both intracellular lipid and secreted FFAs fractions. Notably, intracellular FA proportion of myristic acid (C14:0) was induced in all engineered strains whereas the increase of stearic acid (C18:0) composition was found in extracellular FFAs fraction. Altogether, these overexpressing strains efficiently produced higher lipid production via homeostasis balance on both its lipid synthesis and FFAs secretion.

## Introduction

**Figure 1:**
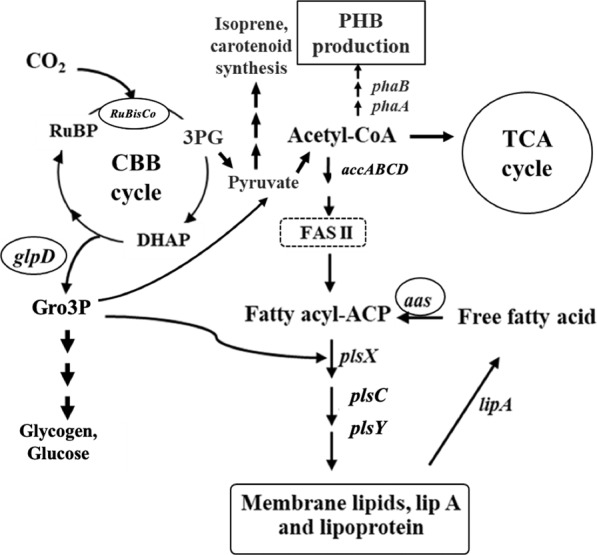
Fatty acid biosynthesis and its neighbouring pathways in *Synechocystis* PCC 6803. Key enzyme-encoding genes in this study (represented in the circle) associated with lipid and fatty acid synthesis, the TCA cycle, the Calvin-Benson-Bassham (CBB) cycle, and free fatty acid (FFA) recycling including *accABCD*, a multi-subunit acetyl-CoA carboxylase gene; *aas*, acyl-ACP synthetase; *glpD*, glycerol-3-phosphate dehydrogenase; *lipA*, a lipolytic enzyme-encoding gene; *phaA*, β-ketothiolase; *phaB*, acetoacetyl-CoA reductase; *plsX*, *plsY* and *plsC*, putative phosphate acyl-transferases; the *RuBisCo* gene cluster, including *rbcLSX*, encoding RuBisCo large, small and chaperone subunits, respectively. The intermediates are: fatty acyl-ACP, fatty acyl-acyl carrier protein; Gro3P, glycerol-3-phosphate; 3PG, 3-phosphoglycerate.

Fatty acids and lipids, which are present at significant levels in cyanobacteria, may serve as crucial precursors for the production of renewable energy carriers, such as biofuel and biodiesel and they are mainly accumulated in terms of phospholipid membranes which are the composition of cell and thylakoid membranes^1^. Many reports have outlined the strength of genetic metabolic engineering approaches to enhance lipid production by genetically engineering the genes involved in lipid biosynthesis and its neighbouring pathways. For lipid biosynthesis in the cyanobacterium *Synechocystis* PCC6803, as shown in Fig. 1, the pathway starts from acetyl-CoA, which is converted to malonyl-CoA catalysed by acetyl-CoA carboxylase (ACC encoded by *acc* gene), and involves multiple steps of fatty acid synthesis II (FAS II) to obtain an intermediate fatty acyl-ACP, which is mainly used as a substrate for phospholipid synthesis and minor alkane production^2^. A genetically modified *Synechocystis* 6803 strain in which alkane production is blocked by deleting *aar*, encoding an acyl-ACP reductase that catalyses the conversion of fatty acyl-ACP to alkane, and together with an overexpression of *aas*, a gene encoding acyl-acyl carrier protein synthetase (AAS), had enhanced lipid level of approximately 1.8-fold^3^. The FFA recycling occurs via the activity of AAS, encoded by *aas*, to recycle the degraded membrane lipids by lipase A, encoded by *lipA*, to intracellular FFAs and which is then reused as a precursor for fatty acyl-ACP production^4,5^. In brief, in the linkage pathway with lipid synthesis, the Calvin-Benson-Bassham (CBB) cycle uses ATP and NADPH obtained from the light reaction to generate sugars^6^. Ribulose-1,5-bisphosphate carboxylase/oxygenase, commonly known as RuBisCO, is the key enzyme in the CBB cycle and plays a crucial rate-limiting role in this pathway. An engineered *Synechocystis* PCC 6803 strain overexpressing RuBisCO, encoded by *RuBisCo* gene operon (*rbcLXS*), exhibited increased RuBisCO activity, growth rate and biomass^7^. Not only the carbon source but also many carbon intermediates are produced within the CBB cycle and indirectly flow to other crucial metabolic processes; for example, the conversion of the intermediate 3-phosphoglycerate (3-PG) to pyruvate and subsequently to acetyl-CoA, which are used in central metabolic pathways^8^. Moreover, dihydroxyacetone phosphate (DHAP) which is one of intermediates in the CBB cycle can be converted to glycerol-3-phosphate (Gro3P) catalysed by glycerol-3-phosphate dehydrogenase (GPD encoded by *glpD* gene)^6^, which is further partly used as a glycerol backbone for lipid molecules. Recent research revealed the enhancement of Gro3P synthesis, which enhanced lipid production in *Synechocystis* PCC6803, by introducing the heterologous *GPD1* gene (encoding glycerol-3-phosphate dehydrogenase) from *Saccharomyces cerevisiae* and the *atf1* gene (encoding diacylglycerol acyltransferase, DGAT) from the oleaginous bacterium *Rhodococcus opacus* PD630^9^. However, Gro3P also generally functions as a substrate for other pathways including starch and glycogen biosynthesis, isoprene and carotenoid synthesis^9–12^.

In this study, we metabolically engineered *Synechocystis* 6803 strain via the integrated aspect on carbon fixation and lipid production by modifying genes related to fatty acid recycling and CBB cycle. There were three engineered strains constructed in this study including OG, OAG and OAGR. The OG strain was *glpD*-overexpressing strain which expectedly enhanced Gro3P product and was partly used as glycerol backbone in lipid molecules. The OAG strain contained double overexpression of *aas_glpD* which probably enhanced both glycerol backbone and fatty acyl-ACP substrate whereas triple-overexpression of *aas_glpD_rbcLXS* genes (OAGR) would synergistically induce RuBisCO function beside OAG. Our findings indicate that the apparent creation of genetically modified strains, in particular OAGR, was achieved to augment both intracellular lipid synthesis and free fatty acids secretion.

## Results

### Construct verification and growth curves of engineered *Synechocystis* PCC 6803 strains

**Figure 2:**
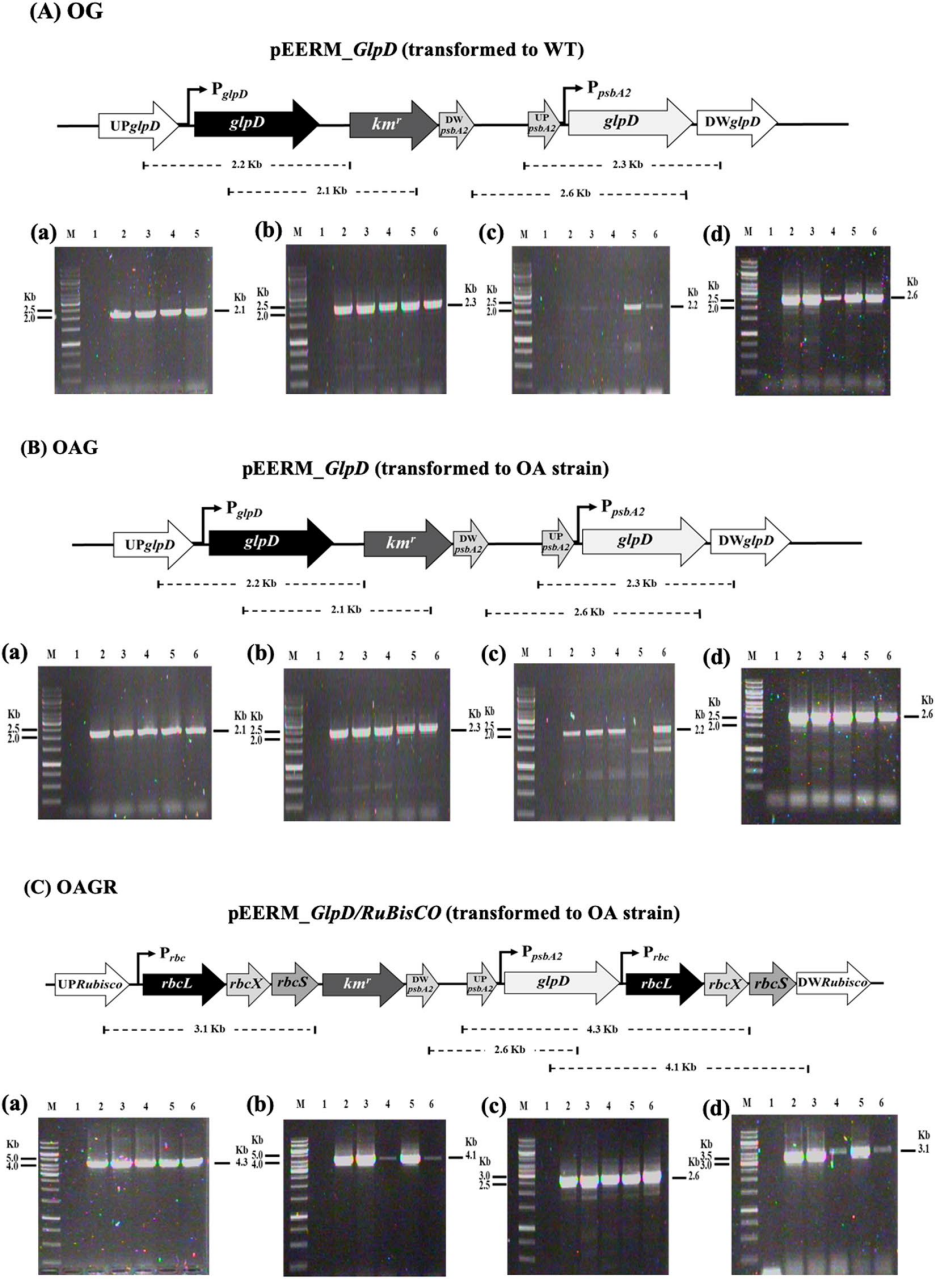
Genomic maps of engineered *Synechocystis* PCC 6803 strains, including the overexpression strains OG (**A**), OAG (**B**), and OAGR (**C**). The specific primers (Supplementary information, Table S1) are used for confirming the integration of each gene into *Synechocystis* genome. The OA strain^3^ was singly recombined with the *aas* gene. The OG and OAG strains were constructed by overexpressing the native *glpd* gene in WT and OA cells, respectively. OAGR was generated by overexpressing *glpd* and a *RuBisCo* cassette in the OA strain. Confirmation of integration was performed using PCR with genomic DNA from WT and engineered strains as the template. Lane M: GeneRuler DNA ladder (FERMENTAS). For (**A**) OG strain; Lane 1: negative control using WT as template (a–d), (a) Lanes 2–5: clone numbers 1 to 4 using Glpd_SF and Km_SR primers, (b) Lanes 2–6: clone numbers 1 to 5 using USpsbA2 and DSglpD primers, (c) Lanes 2–6: clone numbers 1 to 5 using USglpD and bb_cSR primers, and (d) Lanes 2–6: clone numbers 1 to 5 using bb_f1 and Glpd_SR primers. For (B) OAG strain; Lane 1: negative control using WT as template (a–d), (a) Lanes 2–6: clone numbers 1 to 5 using Glpd_SF and Km_SR primers, (b) Lanes 2–6: clone numbers 1 to 5 using USpsbA2 and DSglpD primers, (c) Lanes 2–6: clone numbers 1 to 5 using USglpD and bb_cSR primers, and (d) Lanes 2–6: clone numbers 1 to 5 using bb_f1 and Glpd_SR primers. For (**C**) OAGR strain; Lane 1: negative control using WT as template (a–d), (a) Lanes 2–6: Lanes 2–6: clone numbers 1 to 5 using USpsbA2 and RBC_SR2 primers, (b) Lanes 2–6: clone numbers 1 to 5 using GlpD_SF and DSrubisco primers, (c) Lanes 2–6: clone numbers 1 to 5 using bb_f1 and Glpd_SR primers, and (d) Lanes 2–6: clone numbers 1 to 5 using USrubisco and bb_cSR primers.

**Figure 3:**
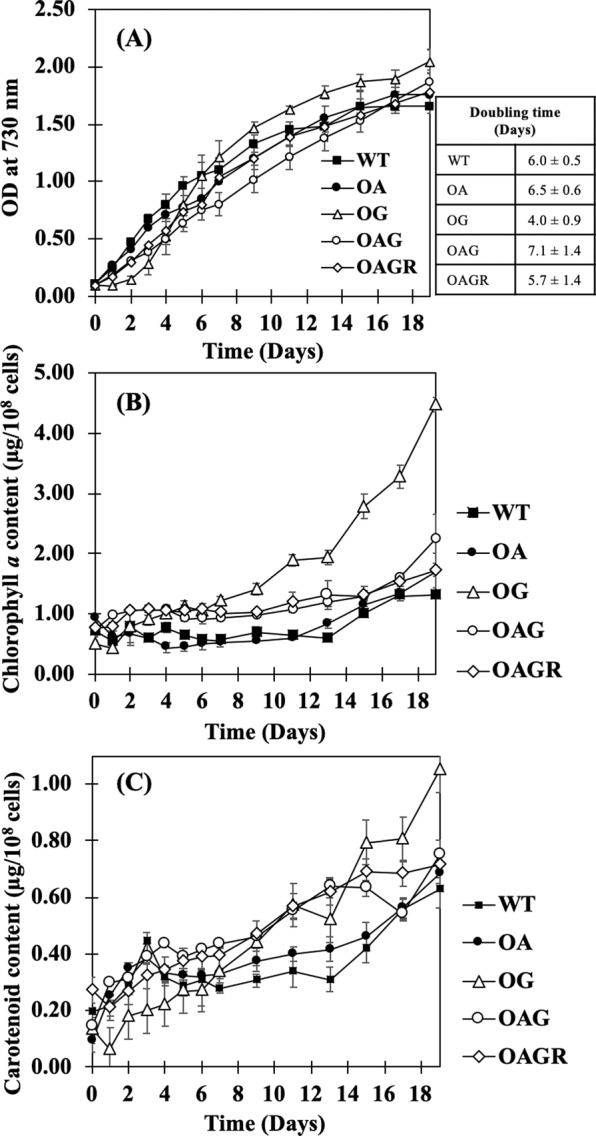
Growth curve (**A**), chlorophyll *a* content (**B**) and carotenoid content (**C**) of the *Synechocystis* PCC6803 wild-type (WT), OA, OG, OAG, and OAGR strains grown in BG_11_ medium for 19 days. The error bars represent standard deviations of means (mean ± S.D., n = 3). Doubling time of growth for each strain is shown in Table at right-handed side of (**A**).

After we successfully obtained all the engineered transformant cells (Table 1, Fig. 2), the strains were verified by monitoring gene location and segregation by PCR using many specific primers (Supplementary information, Table S1). Both wild type (WT) cells and the *aas-*overexpressing strain or OA, obtained from our previous study^3^, were used as hosts for further engineering in this study (Table 1). The OG and OAG strains were generated by the single homologous recombination into the native *glpD* gene (Fig. 2A,B), whereas OAGR was constructed by introducing *glpD* and *rbcLXS* gene fragments into the native *RuBisCo* gene operon (Fig. 2C). The gene integration was confirmed using PCR by each specific pair of primers (Supplementary information, Table S1) as shown in (a)–(d) of Fig. 2A–C. After that, cell growth of the engineered strains was subsequently monitored by comparing with *Synechocystis* PCC 6803 WT. The slight decrease of cell growth was observed in most engineered strains, except OG which apparently had increased cell growth after 6 days of cultivation when compared to WT (Fig. 3A). Doubling time of OG’s cell growth (4 days) was efficiently shortest among all strains studied whereas OAG (double *aas*_*glpD*-overexpressing strain) had longest doubling time. The OG strain apparently showed faster growth when compared to other strains. Moreover, intracellular pigments of chlorophyll *a* and carotenoids (Fig. 3B,C) of OG, OAG and OAGR strains showed the distinctly higher contents when compared to those of both WT and OA strains, in particular OG.

**Table 1:** Strains and plasmids used in this study.

Name	Relevant genotype	Reference
**Cyanobacterial strains**		
*Synechocystis* sp. PCC 6803	Wild type	Pasteur culture collection
OA (OXAas)	*aas, cm*^*r*^ integrated at region of native *aas* gene in *Synechocystis* genome	^3^
OG (OXGlpD)	*glpD, km*^*r*^ integrated at region of native *glpD* gene in *Synechocystis* genome	This study
OAG (OXAas/GlpD)	*aas, cm*^*r*^ integrated at region of native *aas* gene in *Synechocystis* genome *glpD, km*^*r*^ integrated at region of native *glpD* gene in *Synechocystis* genome	This study
OAGR (OXAas/GlpD/RuBisco)	*aas, cm*^*r*^ integrated at region of native *aas* gene in *Synechocystis* genome *glpD, Rubisco; rbcL,rbcX, rbcS, km*^*r*^ integrated at region of native *Rubisco* gene in *Synechocystis* genome	This study
**Plasmids**		
pEERM	P_psbA2_ – *cm*^*r*^; plasmid containing flanking region of *psbA2* gene	^22^
pEERM_*Aas*	P_psbA2_ – *aas - cm*^*r*^; integrated between *Xba*I and *Spe*I sites of pEERM	^3^
pEERM_Km	P_psbA2_ – *km*^*r*^; plasmid containing flanking region of *psbA2* gene	This study
pEERM_pECmKm	P_km_^r^ – *km*^*r*^; integrated at *BamH*I sites of pEERM	This study
pEERM_*GlpD*	P_psbA2_ – *glpD*; integrated between *Xba*I and *Spe*I sites of pEERM_Km	This study
pEERM_*GlpD_RubisCo*	P_psbA2_ – *glpD* and P_*rbcL*_*-RubisCo*; integrated between *Xba*I/*Spe*I and *SpeI/PstI* sites of pEERM_Km, respectively	This study
pJAasCm	P_T7_– *aas-cm*^*r*^; plasmid containing *cm*^*r*^ between the flanking region of *aas* gene	This study

P_psbA2_, strong *psbA2* promoter; *cm*^*r*^, chloramphenicol resistance cassette.

### Productions of intracellular lipids and extracellular free fatty acids (FFAs), and fatty acid (FA) compositions of engineered strains

**Figure 4:**
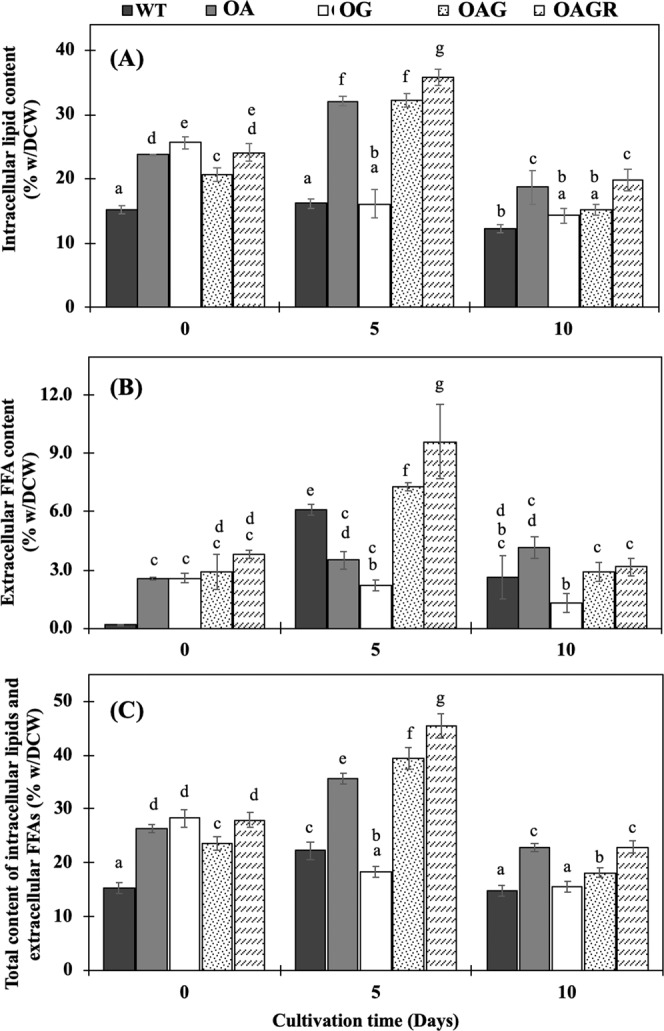
Intracellular lipid content (**A**), extracellular free fatty acid content (**B**) and total contents of both intracellular lipids and extracellular free fatty acids (**C**) in *Synechocystis* PCC 6803 WT, OA, OG, OAG and OAGR strains grown in BG_11_ medium at the start of the experiment (day 0), day 5 and day 10 of cultivation. The error bars represent standard deviations of means (mean ± S.D., n = 3). Means with the same letter have nonsignificant differences at a significance level of *P* < 0.05.

At the start of cultivation (day 0), it was evident that all engineered strains contained significantly higher contents of intracellular lipids compared with WT cells (Fig. 4A). At day 5 of cultivation, the significant increases of intracellular lipid contents were observed in the OA, OAG and OAGR strains, but not in OG which exhibited similar content to that of WT with about 16% w/DCW. The highest content of intracellular lipids was noted at day 5 in OAGR strain, with approximately 35.9% w/DCW, followed by 32.2 and 32.1% w/DCW in OAG and OA, respectively. The certain decreases of lipid contents of all strains were observed at day 10 of cultivation, except OG strain which showed its constant level of intracellular lipid contents between day 5 and day 10 of cultivation. We also checked the intracellular lipid titer (mg/L) and production rate (mg/L/day) which were calculated and shown in Table 2. OA strain had the highest lipid titer and production rate at day 5 with 228.7 mg/L and 45.7 mg/L/day, respectively, whereas OG strain at day 10 had 358.3 mg/L and 35.8 mg/L/day, respectively (Table 2). As a result of faster growth of OG, its lipid content per cell mass (% w/DCW) was almost constant between day 5 and day 10 of cultivation (Fig. 4A).

**Table 2:** Intracellular lipid titers and production rates of engineered *Synechocystis* PCC 6803 strains (means ± S.D., n = 3). Means with the same letter show insignificant differences with a significant level at *P* < 0.05.

Strains	Start (day 0)	Lipid titers (mg/L)		Production rates (mg/L/day)	
		day 5	day 10	day 5	day 10
WT	9.1 ± 0.4^a^	139.8 ± 26.0^d^	137 ± 22.2^d^	28.0 ± 5.2^k^	13.7 ± 2.2^l^
OA	14.6 ± 0.6^c^	228.7 ± 6.0^g^	269.8 ± 17.7^h^	45.7 ± 1.2°	27.0 ± 1.8^k^
OG	14.2 ± 0.5^c^	189.1 ± 37.0^e,d^	358.3 ± 79.2^i^	37.8 ± 7.4^m^	35.8 ± 7.9^m^
OAG	12.8 ± 0.7^b,c^	206.0 ± 13.3^f^	266.3 ± 14.3^h^	41.2 ± 2.7^n^	26.6 ± 1.4^k^
OAGR	14.2 ± 0.9 ^c^	200.5 ± 7.5^f^	344.6 ± 33.9^i^	40.1 ± 1.5^n^	34.5 ± 3.4^m^

Not only intracellular lipid synthesis but also free fatty acid (FFA) secretion contribute towards intracellular FFA balance upon toxic tolerance mechanism of cyanobacterial cells. Results of extracellular FFA content secreted into the medium are shown in Fig. 4B. At day 5, the *Synechocystis* WT strain secreted FFAs into BG_11_ medium at a level of approximately 6.1% w/DCW (Fig. 4B) with its high titer of about 7.3 mg/L (Table 3) when compared to the highest titer from OAGR (7.8 mg/L). The decreases of secreted FFAs levels from OA and OG strains were apparently noted when compared with that of WT (Fig. 4B), as well as their secreted titer (Table 3). Strikingly, OAG and OAGR strains were able to secrete significantly greater amounts of extracellular FFAs at day 5 than WT cells by about 7.3 and 9.6% w/DCW, respectively. In Table 3, the secreted FFA titers and production rates of OA, OG and OAG strains were lower than that of WT at day 5 but higher at day 10, except OG strain. The highest titer and production rate of the secreted FFAs occurred at day 5 and day 10 in OAGR and OA strains, respectively (Table 3). In Fig. 4C, we combined the results of both intracellular lipids and extracellular FFAs contents in order to reveal the capacity of cells based on their lipid synthesis and FFAs secretion, although FFA is only a component of lipid molecule. The highest capacity of triple overexpressing strain, OAGR, was evident on both intracellular lipid synthesis and FFA secretion, especially at day 5 of cultivation (Fig. 4C).

**Table 3:** Extracellular free fatty acid (FFA) titers and production rates of the engineered *Synechocystis* PCC 6803 strains (means ± S.D., n = 3). Means with the same letter show insignificant differences with a significant level at *P* < 0.05.

Strains	Extracellular FFA titers (mg/L)			Production rates (mg/L/day)	
	Start	day 5	day 10	day 5	day 10
WT	0.04 ± 0.01^a^	7.3 ± 0.9^f,g^	7.2 ± 0.5^f,g^	1.46 ± 0.19^m^	0.72 ± 0.05^j^
OA	0.63 ± 0.04^c^	3.7 ± 0.3^e^	9.0 ± 0.2^i^	0.75 ± 0.06^j^	0.90 ± 0.02^k^
OG	0.56 ± 0.01^b^	3.9 ± 0.9^e^	4.6 ± 0.5^e^	0.78 ± 0.18^j,k^	0.46 ± 0.05^n^
OAG	0.66 ± 0.11^c^	7.0 ± 0.4^f^	7.7 ± 1.9^g^	1.40 ± 0.07^k^	0.77 ± 0.19^j,k^
OAGR	0.90 ± 0.10^d^	7.8 ± 0.6^g^	8.2 ± 0.3^h^	1.55 ± 0.13^m^	0.82 ± 0.03^l,k^

**Figure 5:**
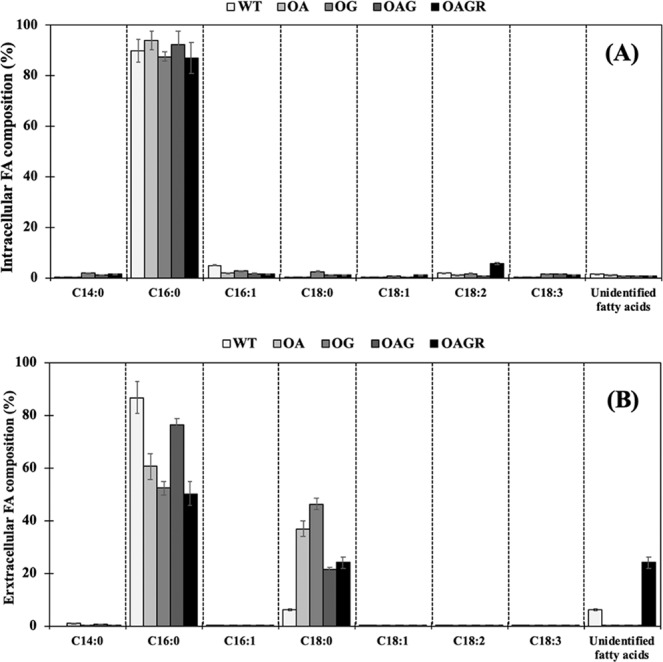
Intracellular (**A**) and extracellular (**B**) FA compositions (%) analysed by a GC instrument in *Synechocystis* PCC6803 WT and engineered strains grown in BG_11_ medium at day 10 of cultivation. The error bars represent standard deviations of means (mean ± S.D., n = 3).

The fatty acid (FA) compositions from extracted cells (intracellular FAs) and supernatant (extracellular FAs) fractions of all strains were analysed by GC instrument. The results are shown as percentage of FA compositions (Fig. 5). The dominant FA in *Synechocystis* cells was palmitic acid (C16:0) in the range of 87–93% (Fig. 5A). The genetically modified strains in this study could increase C16:0 FA, especially OA and OAG strains. On the other hand, the decrease of stearic acid (C18:0) was markedly detected in all engineered strains when compared to that of WT whereas the increased ratio of linoleic acid (C18:2) were apparently noted in the OAGR strain. It was interesting that myristic acid (C14:0) was induced in the engineered strains, especially OG (Fig. 5A). In Fig. 5B, the FA composition in secreted FFAs fraction was depicted. The dominant C16:0 was also extracellularly found in all strains. The increased proportion of C18:0 by about 21–46% were secreted from all engineered strains when compared to that from WT (6%). Thus, the FFAs secretion from engineered *Synechocystis* cells mainly consisted of palmitic acid (C16:0) and stearic acid (C18:0) (Fig. 5B).

### Transcript levels of genes involved in CBB cycle and fatty acid biosynthetic pathway

**Figure 6:**
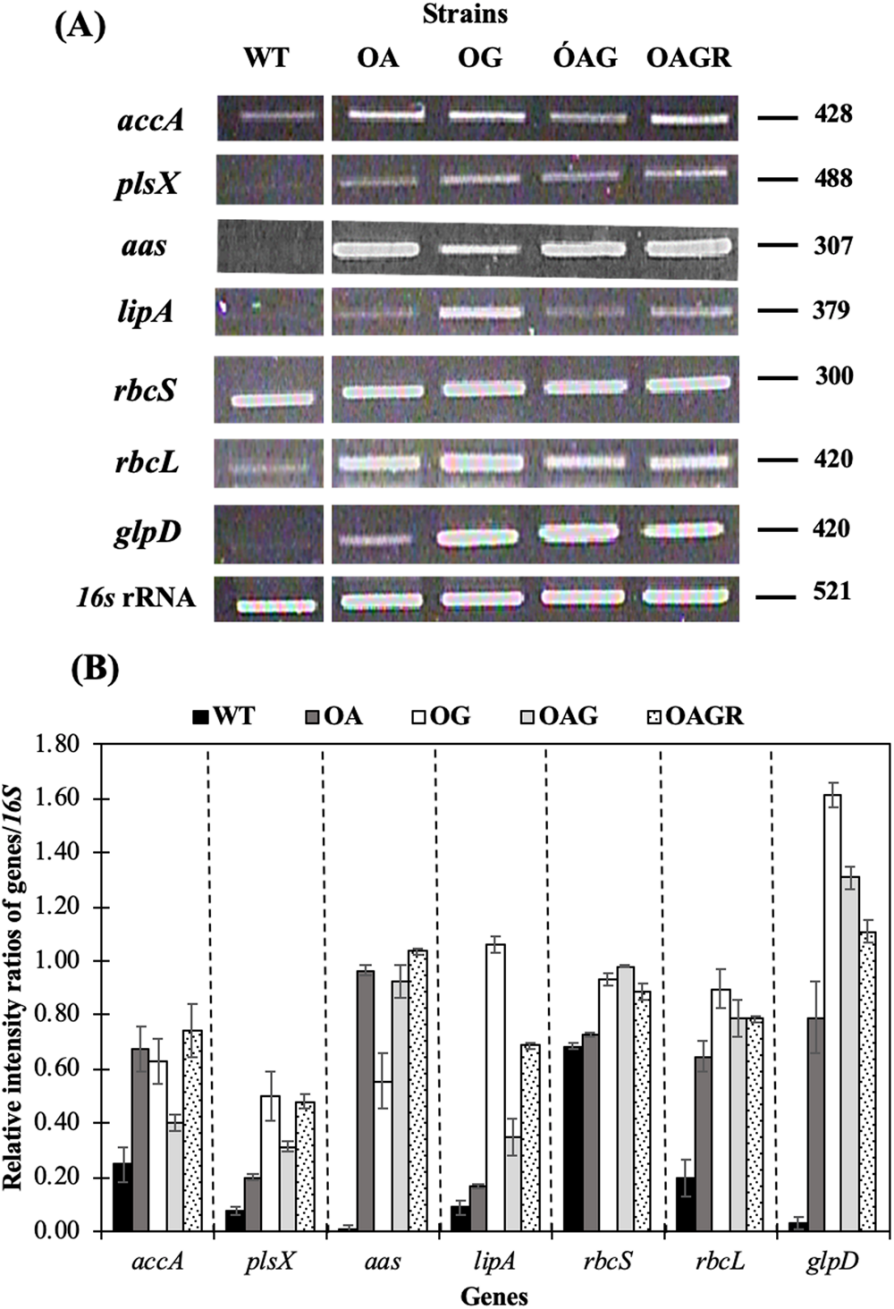
The transcript levels (**A**) and relative intensity ratios (**B**) of the *accA*, *plsX*, *aas*, *lipA*, *rbcS*, *rbcL, glpD* and *16S* rRNA genes of *Synechocystis* PCC 6803 WT, OA, OG, OAG, and OAGR strains grown in BG_11_ medium at day 5 of cultivation. The error bars represent standard deviations of means (mean ± S.D., n = 3). The cropped gels (in **A**) were taken from the different gels, and each cropped gel, between WT and OA bands, was taken from the same gel cutting out the non-related band of other sample as shown in Supplementary Information.

The five day-cell culture of each strain was used to determine the transcript amounts of genes involved in CBB cycle, fatty acid and lipid biosynthesis and lipid degradation (Fig. 1) including *accA, plsX, aas, lipA, rbcS, rbcL* and *glpD* genes by RT-PCR using various specific pairs of primers (Supplementary information, Table S1). For genes related to fatty acid synthesis, the transcript levels of *accA* gene, encoding acetyl-CoA carboxylase subunit A, were obviously up-regulated in engineered strains, OA, OG, OAG and OAGR, when compared to WT (Fig. 6). Similarly, transcript levels of *plsX* gene, encoding putative acyltransferase for phospholipid synthesis^13^, were increased in engineered strains (Fig. 6B). When compared among four engineered strains, OAG gave lowest level of *accA* transcript whereas OA had lowest level of *plsX* transcript. In addition, the transcript levels of *aas* gene encoding acyl-ACP synthetase related to FFAs recycling, were highly induced in all engineered strains, even in OG strain when compared with WT. For phospholipid degradation, the increased transcript levels of *lipA*, encoding lipase A, were also observed, particularly in OG and OAGR strains, when compared to WT. The transcript levels of *rbcL* and *rbcS*, belonging to *RuBisCo* gene cassette, were significantly increased not only in OAGR strain but also in other engineered strains (Fig. 6B). Finally, transcript levels of *glpD* gene were higher in all engineered strains compared to WT.

## Discussion

In this study, we highlight our results from genetically modified strains of cyanobacterium *Synechocystis* PCC 6803 with enhanced capacity on their lipid production via the synergistic integration of carbon fixation reaction (or CBB cycle) and FFA recycling. Our previous reports demonstrated the increased lipid levels in engineered *Synechocystis* by modifying genes directly associated with fatty acid and phospholipid synthesis, including *plsX, plsC, accDBCA*, and FFA recycling including *aas* using metabolic engineering approach^3,13^. We previously suggested that carbon source supplementation, particularly acetate addition, could induce the intracellular lipid content up to 39.1% w/DCW in *PlsC_PlsX*-overexpressing *Synechocystis* cells^13^ (reviewed in Table 4). In this study, we then created the engineered strain with itself overproduced C-source via carbon fixation reaction by overexpressing *RuBisCo* gene operon (*rbcL*, *rbcX* and *rbcS*), thereby gaining more 3PG intermediate which partially changed to acetyl-CoA, the initial substrate required for fatty acid and phospholipid synthesis (Fig. 1). Moreover, recent overexpression of *glpD* gene encoding glycerol-3-phosphate dehydrogenase (GlpD), a crucial link between carbohydrate, pigment and lipid biosyntheses^9,10^ could increase glycerol backbone in terms of Gro3P which could partly contribute to phospholipid synthesis as well. On the other hand, Farmer and Liao (2001) reported that Gro3P could be also converted to pyruvate intermediate which was sequentially utilized for isoprene and carotenoid synthesis. Moreover, in our previous work, *Synechocystis* cells with overexpressing *aas* gene significantly increased their intracellular lipid level under normal growth condition^3^. To develop more efficient strains in this study, we recombined the *glpD* and/or *RuBisCo* with *aas* overexpression which consequently generated OG, OAG and OAGR strains. Our results demonstrated that *glpD*-overexpressing *Synechocystis* strain (or OG) had the highest increase of cell growth (or biomass) and intracellular pigments, including chlorophyll *a* and carotenoids (Fig. 3). The *glpD* overexpression of OG had higher induction effect on chlorophyll *a* content which tightly correlated with the light reaction of photosystems^14^. When cells gained higher CBB cycling by *glpD* overexpression, more ATP and NADPH generated from the light reaction would be needed. Thus, changes in chlorophyll *a* content may reflect changes in photosystem stoichiometry^14^. The time required to double population of cells or doubling time also confirmed that OG strain had fastest growth among all strains with the doubling time of 4 days. Our results suggested that the higher Gro3P product observed in OG strain might metabolically flow through glycolysis and TCA cycle which are crucial for generating energy for higher cell growth. The triple *aas*_*glpD*_*RuBisCo* overexpression (or OAGR) had 5.7 days of doubling time which slightly grew faster than WT (6 days of doubling time). However, the previous study suggested that overexpression of RuBisCO driven under *psbA2* promoter could slightly increase the photosynthesis and biomass yield of *Synechocystis* PCC 6803^7^. Only few studies have reported the use of Gro3P (glycerol-3-phosphate), a major product of the *glpD*-catalysed reaction, where Gro3P could ultimately flow to gluconeogenesis, glycolysis and lipid synthesis^9^. The strain overexpressing *aas*_*glpD_Rubisco* (OAGR strain) gave the highest level of intracellular lipid accumulation of approximately 35.9% w/DCW at day 5 of cultivation (Fig. 4), although its lipid production rate was 40.1 mg/L/day which was lower than that of OA with 45.7 mg/L/day due to its lowered biomass at day 5 (Table 2). Our results indicated that the intracellular lipid titer and production rate have a tight connection with cell biomass. Moreover, we proposed that Gro3P, the product of GPD activity, preferred to mainly flow to carbohydrate and pigment syntheses as supported by the increase of cell growth and intracellular pigment contents (Fig. 3). The reduced intracellular lipid content of the *glpD*-overexpressing strain (OG) (Fig. 4A) was reasonable due to the increased cell growth or biomass. This result was also supported by *lipA* transcript increase (Fig. 6), relating to phospholipid degradation into FFAs which were further utilized as energy for cell growth inside OG cells. We also demonstrated the high intracellular lipid content from our metabolic engineering design compared with previously reported works which focused on carbon supplementation (Table 4). The adjustment of nutrient composition in BG_11_ medium together with CO_2_ bubbling might be useful to further improve lipid production of our genetically-modified strains.

**Table 4:** Enhancement of intracellular lipid contents in microorganisms.

Microorganisms	Strains (wild type or genetically modified)	Modified condition for production	Lipid contents (%/DCW)	References
**Yeast**				
*Saccharomyces cerevisiae*	Overexpression of *FAS1, FAS2*, and *ACC1* genes	synthetic liquid media	17.0%	^28^
	Expression of heterologous *DGAT1* gene from *Brassica napus*	synthetic liquid media containing 2%(w/v) glucose, 2% (*w*/*v*) galactose and 1% (*w*/*v*) raffinose	28.0%	^29^
	Wild type	Nitrogen deprivation (5 mM (NH_4_)_2_SO_4_)	Over 20%	^30^
	Expression of *ACC* from an oleaginous yeast, *Lipomyces starkeyi*	YPD medium	45%	^31^
**Microalgae**				
*Chlamydomonas reinhardtii*	Expression of heterologous *DGAT2* gene from *Brassica napus*	TAP medium	18.76%	^32^
*Nannochloropsis salina*	Expression of heterologous *AtWRI1* gene from *Arabidopsis*	L1 enriched artificial seawater adjusted to pH 8.0	36.5% (normal) and 44.7% (osmotic stress)	^33^
**Bacteria**				
*Rhodococcus rhodochrous*	Wild type	LB medium with 20 mg/L of glucose	50%	^34^
*Escherichia coli*	Overexpression of four multiple subunits encoding ACC	LB medium:10 g of tryptone, 1 g of yeast extract, 5 g of NaCl	6 fold increase	^35^
**Cyanobacteria**				
*Synechococcus* sp.HS01	Wild type	Mixotrophic cultivations including CO_2_, glucose with CO_2_ and ostrich oil with CO_2_	12.3, 16 and 32%, respectively	^36^
*Synechocystis* sp. PCC 6803	Overexpresion of *plsX/plsC* genes	BG_11_ medium	24.3%	^13^
	Overexpresion of *plsX/plsC* genes	BG_11_ medium with 0.4% (w/v) acetate supplementation	39.1%	^13^
	Overexpression of *aas* gene	BG_11_ medium (4 days)	34.5%	^3^
	Triple overexpression of *aas/glpD/RuBisCo*	BG_11_ medium (5 days)	35.1%	This study

In this study, we also showed the results of free fatty acid (FFA) secretion (Fig. 3B). Recent reports revealed that the excessive level of intracellularly synthesized FFAs cause cell damage, i.e. unsaturated FAs can generate significant levels of reactive oxygen species (ROS) inside cells^15^ and FFAs could intercalate into both cell and thylakoid membranes which caused destabilization of both proteins and photosynthesis^16–18^. One of detoxification mechanisms is the FFAs secretion out of cells in order to reduce excessive FFA toxicity inside cells. *Synechocystis* cells have another mechanism to balance lipid metabolites or respond to changed environment by hydrolyzing phospholipid membrane into intracellular FFAs via the activity of lipase A enzyme encoded by *lipA*^5^. Our results demonstrated the increase of secreted FFAs content at day 5 of two engineered strains, OAG and OAGR, with up to 7.8 and 9.8% w/DCW, respectively compared with WT (6.0% w/DCW). The significant increase of both intracellular lipids and extracellular FFAs was observed in OAGR strain. We found that there was a variable correlation between the *lipA* transcript levels (Fig. 6) and extracellular FFA contents (Fig. 4B) among the engineered strains. For example, OAGR strain had high *lipA* transcript level and high extracellular FFA, whereas OG strain had high *lipA* transcript level but low extracellular FFA. This might suggest that the enhanced amount of intracellular FFAs via phospholipid degradation could be an internal regulatory mechanism of the utilization of FFA, i.e. being used intracellularly as energy source for cell growth including its recycling or being secreted into the medium. In this respect, OAGR strain was found to be most efficient for both lipid synthesis and FFA secretion (Fig. 4) amendable for commercial application. In addition, we analysed fatty acid composition of internal lipid and secreted FFAs fraction (Fig. 5). Palmitic acid (C16:0) was the dominant FA in *Synechocystis* cells which is in agreement with previous studies in wild type^3,13,19^. It is interesting that myristic acid (C14:0) was also found in all constructed strains at day 10. In a previous study, *in vitro* acyl-ACP synthetase (AAS) of bacterial *Vibrio harveyi* preferred C14:0 as one of the substrates for fatty acyl-ACP synthesis^20^. The high myristic acid levels were mainly found to be unique to *Cyanothece* sp., aided by the enzyme 1-acyl-sn-glycerol-3-phosphate acyltransferase with high specificity towards the 14:0-acyl-carrier protein^21^. Comparisons with genome databases such as Cyanobase revealed that this enzyme in *Cyanothece* PCC8801_1274 shares 59.6% identity with *Sll1848* (*PlsC*) of *Synechocystis* PCC 6803. Coincidentally, our previous report demonstrated that *Sll1848* overexpression led to increased lipid production in cells^13^. Moreover, our findings indicate the increased ratio of stearic acid (C18:0) in extracellular FFAs fraction from all engineered strains (Fig. 5B). In this respect, it should be noted that the structure of C18:0 is ordinarily more hydrophobic which can easily diffuse through cell membrane and thylakoid membranes. On the other hand, we also demonstrated the transcript levels of all the overexpression strains determined by RT-PCR (Fig. 6). Our results indicated that the induced transcript amounts of engineered strains were *accA* and *plsX* genes associated with fatty acid synthesis. The result of double *glpD* and *aas* overexpressing strain (or OAG) showing lower amount of *accA* transcript among engineered strains might indicate the feedback inhibition of fatty acyl-ACP to acetyl-CoA carboxylase (ACC) in fatty acid synthesis. However, triple overexpression by OAGR strain could increase an *accA* transcript level by about 2-fold compared to OAG strain suggesting that the OAGR strain could overcome feedback inhibition effect. Notably, our results also indicated the up-regulated transcript levels of *lipA*, encoding the lipolytic enzyme associated with phospholipid hydrolysis, in all engineered strains compared to WT. It was interesting that the *rbcS* transcript level was apparently higher than *rbcL* transcript in WT with low level of *glpD* gene expression (Fig. 6). All engineered strains significantly enhanced the expression of *RuBisCo* and *glpD* genes involved in CBB cycle.

In conclusion, our metabolically engineered *Synechocystis* strains were successfully constructed via integrative expression of *aas*, encoding acyl-ACP synthetase involved in FFA recycling, combined with *RuBisCo* and *glpD* genes in the Calvin-Benson-Bassham cycle. The genetically engineered *Synechocystis* strains in this study could be classified into three categories according to the desired purposes. First, the modified strain with best performance on internal lipid balance belongs to OAGR strain, as evident by highest lipid and FFA contents at day 5 of cultivation. Second, with regard to biotechnological productivity, OA is the best strain which prominently achieves highest lipid titer and production rate at day 5 of cultivation, Finally, for the purpose of increasing the biomass for lipids and other metabolites production, OG strain is the promising candidate. However, to further enhance the lipid productivity of selected genetically engineered strains, the stressed environment should be imposed in order to alter their metabolic flux and balance towards lipid synthesis.

## Methods

### Strains and culture conditions

The host *Escherichia coli* DH5α strain was propagated on Luria Bertani (LB) agar plates and in liquid broth containing 35 µg/mL kanamycin (Km) and 35 µg/mL chloramphenicol (Cm) at an optimum temperature of 37 °C. The unicellular cyanobacterium *Synechocystis* sp. PCC6803 (*Synechocystis*) strain was grown in BG_11_ medium at a culture temperature of 28 °C with continuous illumination of approximately 50 µmol photons/m^2^/s. The *aas*-overexpressing *Synechocystis* (OX*Aas*) strain was obtained in our previous study^3^, hereafter OA in this study. Other engineered strains were OG, OAG and OAGR strains, all of which were constructed in this study (Table 1). The cells were routinely cultured in BG_11_ medium containing both 35 µg/mL kanamycin and 35 µg/mL chloramphenicol.

### Construction of recombinant plasmids

All strains and plasmids constructed in this study are listed in Table 1. Initially, we modified the original pEERM expression vector^22^ by replacing the antibiotic *Cm*^*r*^ cassette gene with the *Km*^*r*^ cassette gene to obtain the pEERM_Km vector, which was subsequently used as a cloning and expression vector. The recombinant pEERM_*GlpD* plasmid was constructed by inserting a homologous *glpD* gene fragment, amplified by PCR using the GlpD_F and GlpD_R primers (Supplementary information, Table S1) and genomic DNA of *Synechocystis* as a template, into the pEERM_Km vector between the *Xba*I and *Bcu*I restriction sites. Additionally, the recombinant pEERM_*GlpD_RubisCo* (Table 1) was constructed by introducing a cassette fragment containing the *rbcL*, *rbcX* and *rbcS* genes, obtained from PCR using the RBC-F and RBC-R primers, into the pEERM_*GlpD* vector between the *Bcu*I and *Pst*I restriction sites. The pJAasCm vector (Table 1) was created by ligating the *aas* gene fragment amplified by PCR using the aas_F3 and aas_R3 primers (Supplementary information, Table S1) into the pJET1.2 blunt cloning vector whereas the antibiotic *Cm*^*r*^ cassette gene fragment was amplified by the cm_F and cm_R primers (Supplementary information, Table S1) using the pEERM plasmid as the template. The *Cm*^*r*^ fragment was subsequently inserted into the *Avr*II restriction site of the *aas* gene ligated into the pJET vector.

### Natural transformation of recombinant plasmids into Synechocystis cells

To prepare host cells, *Synechocystis* PCC 6803 wild type (WT) and OA strains were initially grown in BG_11_ liquid medium until the optical density at 730 nm (OD_730_) reached between 0.3 and 0.5. 25 mL of cultured cells were harvested by centrifugation at 6,000 rpm (4,025 × *g*) for 10 min. Subsequently, the pelleted cells were washed and harvested again by centrifugation at the same speed for 10 min before being resuspended in 0.5 mL of fresh BG_11_ medium for cell condensation. To construct the OG strain, 1 µg of the recombinant pEERM_*GlpD* plasmid was added to the condensed WT cells and incubated at 28 °C for 6 h, and the tubes were inverted every 2 h. Next, the plasmid-cell mixture was spread on a 0.45-µm sterile nitrocellulose membrane placed over a BG_11_ agar plate overnight, and then, the membrane was transferred to a new BG_11_ agar plate containing 35 µg/mL kanamycin. Similarly, other recombinant plasmids were separately transformed into condensed host cells of WT and OA strains, and each mixture was spread on a 0.45-µm sterile nitrocellulose membrane placed over a BG_11_ agar plate overnight. Then, the membrane was transferred to BG_11_ agar containing the specific antibiotic type corresponding to the expression system, such as 35 µg/mL kanamycin alone or combined with 35 µg/mL chloramphenicol. After 2–3 weeks of incubation, the surviving colonies were picked and further checked for the corresponding gene location and segregation by PCR using specific pairs of primers (Supplementary information, Table S1).

### Measurement of cell growth and pigment content

*Synechocystis* cell stock cultured in BG_11_ medium to mid-log phase of cell growth was harvested and subsequently diluted with fresh BG_11_ medium at an OD_730_ of approximately 0.1 before starting the cultivation experiment. Cell growth was periodically monitored by a spectrophotometer at OD_730_. For measurement of intracellular pigment content, including chlorophyll *a* (chl *a*) and carotenoids, 1 mL of cultured cells were harvested by centrifugation at 12,000 rpm and the pigments were extracted using the methods described by^23,24^. N,N-dimethylformamide (DMF), and the absorbance of the DMF extract were spectrophotometrically measured at 461, 625 and 664 nm. The values were then calculated and normalized to cell number to obtain final values corresponding to 1.0 × 10^8^ cells. Dry cell weight (DCW) was measured by incubating the harvested cells at 60–70 °C until a constant dry weight was obtained.

### Lipid extraction

The cultured cells started at OD730 of 0.1 cultured in 100 mL of fresh BG_11_ medium. Cultured cells (approximately 10–50 mL) of each strain were harvested by centrifugation at 6,000 rpm (4,025 × *g*) for 10 min at days 0 (start), 5 and 10 for lipid extraction. Lipids extracted from the pellet fraction represented intracellular lipids, whereas extracellular FFAs were obtained from the supernatant fraction. In this experiment, the lipid extraction method was slightly modified from the Bligh and Dyer method^25^. A 1-mL chloroform (CHCl_3_)/methanol (CH_3_OH) solution mixture with a ratio of 2:1 was added to harvested fractions, followed by incubation at 37 °C on a shaker for 2 h. After addition of 0.5 mL of 0.88% potassium chloride (KCl) and centrifugation at the same speed for 5 min, the aqueous phase was removed, whereas the lower organic phase was collected. Then, the lipid-dissolving solvent in the lower phase was evaporated at 70 °C.

### Spectrophotometric method for determination of total lipid content

The total lipid content was determined by the potassium dichromate oxidation method^26^. K_2_Cr_2_O_7_ (0.18 M, 0.5 mL) and concentrated sulfuric acid were serially added into the lipid extract as described above. The reaction mixture was boiled at approximately 105 °C for 30 min. After cooling the mixture to room temperature, 0.5 mL of distilled water was added, and the absorbance at 600 nm (Abs600) was spectrophotometrically measured. Canola oil was used as a commercial standard control sample. The lipid content is represented as % w/DCW.

### Analysis of fatty acid components

The fatty acid compositions were analysed using a gas chromatography (GC) instrument. The modified method according to^27^ was used to generate fatty acid methyl esters (FAMEs). A 1-mL mixture of 5% hydrochloric acid in MeOH solution was added to the tube containing extracted lipids. The reaction tube was further heated at 85 °C for 2 h and cooled to room temperature. Subsequently, distilled water (1 mL) was added, and the mixture was vortexed for a few seconds. After the addition of hexane solution (0.5 mL) into the reaction tube, centrifugation at 6,000 rpm (4,025 × *g*) was performed for 5 min to collect the hexane fraction in the GC vial tube. Additional hexane (0.5 mL) was added, and the mixture was centrifuged again to collect the hexane phase and pooled with the previous hexane extract. Compositions of fatty acids in the hexane phase were analysed by GC and interpreted by comparison with standard equations. The commercial standard of fatty acid mixtures (F.A.M.E., mixed C8-C24, MERCK KGaA, Germany) was prepared as a control. The calculated composition of fatty acids is presented as the percentage of each fatty acid component over the total amount of fatty acids.

### Reverse transcription-polymerase chain reaction (RT-PCR)

TRIzol reagent (INVITROGEN, CA) was used to isolate total RNA from harvested cells. Before converting to cDNA using the SuperScript III First-Strand Synthesis Kit (INVITROGEN, CA), isolated total RNAs were treated with RNase-free DNAseI (FERMENTAS, MA) to remove contaminant genomic DNA. Then, the generated cDNAs were used as templates for PCR amplification of genes involved in the CBB cycle, lipid biosynthesis and some neighbouring pathways, including *rbcL*, *rbcS*, *glpD*, *accA*, *aas*, *plsX*, and *lipA*, with *16s* rRNA as a reference, using the corresponding RT-PCR primers (Supplementary information, Table S1). The PCR conditions consisted of 95 °C for 3 minutes, followed by proper cycles for each gene at 95 °C for 30 seconds, primer melting temperature (T_m_) for 30 seconds and 72 °C for 30 seconds, and then a final extension at 72 °C for 5 minutes. Proper cycles and T_m_ of each pair of primers are shown in Supplementary information (Table S1). The PCR products were verified by 1.2% (w/v) agarose gel electrophoresis. Quantification of band intensity was carried out using Syngene Gel Documentation (SYNGENE, Frederick, MD).

## Supplementary information

Supplementary Data.

## Data Availability

The data that support the findings of this study are available within the article and its supplementary files or from the corresponding author upon reasonable request.
