# Supplementary material for: Synechocystis sp. PCC 6803 overexpressing genes involved in CBB cycle and free fatty acid cycling enhances the significant levels of intracellular lipids and secreted free fatty acids
Source: Sci Rep. 2020 Mar 11;10:4515. doi: 10.1038/s41598-020-61100-4 (PMC7066134; doi:10.1038/s41598-020-61100-4)

Title: *Synechocystis* sp. PCC 6803 overexpressing genes involved in CBB cycle and free fatty acid cycling enhances the significant levels of intracellular lipids and secreted free fatty acids

Kamonchanock Eungrasamee[1], Aran Incharoensakdi[1], Peter Lindblad[2], Saowarath Jantaro[1*]

[1] Laboratory of Cyanobacterial Biotechnology, Department of Biochemistry, Faculty of Science, Chulalongkorn University, Bangkok, 10330, Thailand

[2] Microbial Chemistry, Department of Chemistry – Ångström, Uppsala University, Box 523, SE-75120, Uppsala, Sweden

* Corresponding author, E-mail address: saowarath.j@chula.ac.th, Tel. number: 66-(0)2-218-5431, Fax number: 66-(0)2-218-5418

Supplementary information Table S1 Primers used in this study

Name	Sequence (5' to 3')	Purpose of primer	Target gene/ Expected size	PCR Cycles /T_m	Reference
Km_FBamHI	TAGAGAGGATCCACGGTTGATGAGAGCTTTGTTGTA	PCR for *km*^{r}	1122		This study
Km_FAclI	TAGAGAAACGTTACGGTTGATGAGAGCTTTGTTGTA	PCR for *km*^{r}			This study
Km_RBamHI	TAGAGAGGATCCTGTGTCTCAAAATCTCTGATGTTAC	PCR for *km*^{r}			This study
aas_F3	TAGAGATCTAGAGTGGACAGTGGCCATGGCGC	PCR for *aas*	2091		[3]
aas_R3	TACAGAACTAGTTTAAAACATTTCGTCAATTA	PCR for *aas*			[3]
GlpD_F	TAGAGATCTAGAATGCGTAATTTCCCAGAAATCC	PCR for *glpD*	1668		This study
GlpD_R	TAGAGAACTAGTTCAGTGGAGACAATAGTCGGGA	PCR for *glpD*			This study
RBC_F	TAGAGAACTAGTCATTGCCATAAGTAAAGGCATCC	PCR for *RubisCo*	2815		This study
RBC_R	TAGAGACTGCAGTAATTGACAATTGACAATTCCCCAC	PCR for *RubisCo*			This study
cm_F	TAGAGACCTAGTCGAGTTGATCGGGCACGTAA	PCR for *cm*^{r}	905		This study
cm_R	TAGAGACCTAGTCAGCTCGAGGCTTGGATTCT	PCR for *cm*^{r}			This study
bb-cSR	GATATATCAACGGTGGTATATCCA	Sequencing			This study
RBC_SR2	GGTTTGTGGACAATAAAGCTT	Sequencing			This study
GlpD_SR	GCGGTGGTCCGTGGTGCCAAT	Sequencing			This study
GlpD_SF	ATATGTGGAAGCGTTTGTGGATAAA	Sequencing			This study
Km_SF	CGTCATCAAAATCACTCGCA	Sequencing			This study
Km_SR	TGATGCATGGTTACTCACCACTGCG	Sequencing			This study
UUSpsbA2	CACTCAGATAGGAGCCATCTTGC	Colony PCR			This study
USpsbA2	CTTTAGCGTTCCAGTGGATA	Colony PCR			This study
DSpsbA2	GCGATCGCCTTGGCAAAACAACTA	Colony PCR			This study
DDSpsbA2	CCCGTAGTTGTTCAATGATGATGAT	Colony PCR			This study
USrubisco	GCAACCCCTGATTAGCTTTGCG	Colony PCR			This study
DSrubisco	CCGGAATACTCCCCTGGGAA	Colony PCR			This study
USGlpD	CCTACTGCGGGAAGCCTACGCCG	Colony PCR			This study
DSGlpD	CTTAACCGTTGGCTATGGCGACAG	Colony PCR			This study
bb_f1	AGTTAGCCGTAGTTAGCCC	Colony PCR			This study
RTglpD_F420	GAATATGCGGAACGGTTAGAT	RT-PCR for *glpD*	420	26 cycles	This study
RTglpD_R420	GCGGTGGTCCGTGGTGCCAAT	RT-PCR for *glpD*		/53.5 °C	This study
RTrbcL_F420	GGTTTTAAGGCTCTGCGGGC	RT-PCR for *rbcL*	420	23 cycles	This study
RTrbcL_R420	GATGATGGGGGTGCCAATTTCCT	RT-PCR for *rbcL*		/58 °C	This study
RTrbcS_F300	CAAGCCGTTCTCAGGATCCAG	RT-PCR for *rbcS*	300	22 cycles	This study
RTrbcS_R300	AGGGAAATTATCGACTTCGGCTA	RT-PCR for *rbcS*		/56 °C	This study
RTlipA_F379	TTGGCGGAGCAAGTGAAGCAAT	RT-PCR for *lipA*	379	25 cycles	[3]
RTLipA_R379	CATGGACCAGCACAGGCAAAAT	RT-PCR for *lipA*		/55.1 °C	
RTaccA_F428	ATGCACGGCGATCGAGGAGGT	RT-PCR for *accA*	428	27 cycles	[3]
RTaccA_R428	TGGAGTAGCCACGGTGTACAC	RT-PCR for *accA*		/58.2 °C	
RT16sRNA_F521	AGTTCTGACGGTACCTGATGA	RT-PCR for *16s*	521	13 cycles	[3]
RT16sRNA_R521	GTCAAGCCTTGGTAAGGTTAT	RT-PCR for *16s*		/56 °C	
RTaas_F307	GTGGTTTATCGCCGATCAAG	RT-PCR for *aas*	307	28 cycles	[3]
RTaas_R307	TTCCTGGCGGGGAACGGGAG	RT-PCR for *aas*		/54.5 °C	
RTPlsX_F	AAGGGGTGGTGGAAATGGAA	RT-PCR for *PlsX*	488	27 cycles	[13]
RTPlsX_R	AAGTAGGTCCCTTCCTTCGG	RT-PCR for *PlsX*		/52.7 °C	

Supplementary information Figure S1

The full-length of gel from RT-PCR products of each gel was shown as followings. In each gel picture, we cropped gel by cutting out the non-related bands, Lane 2, of other samples (WTd).

accA, 58.2C, 27 cycles

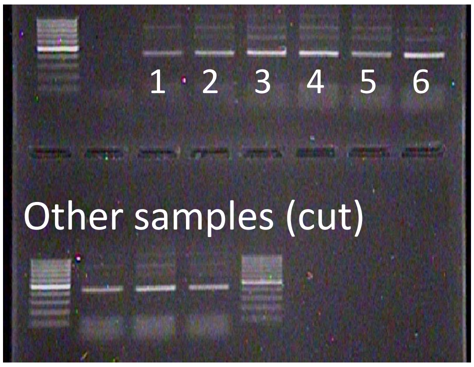

Lane
1: WT
2: WTd (cropped)
3: OA
4: OG
5: OAG
6:OAGR

lipA 55.1 C, 25 cycles

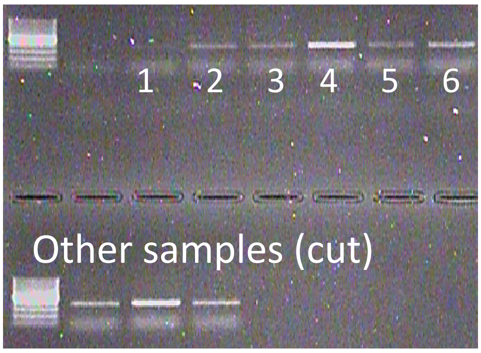

plsX, 52.7C, 27 cycles

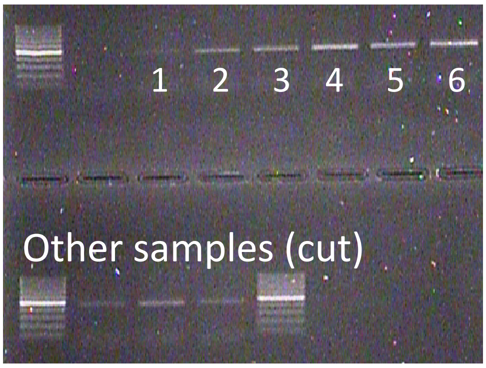

rbcS 56 C, 22 cycles

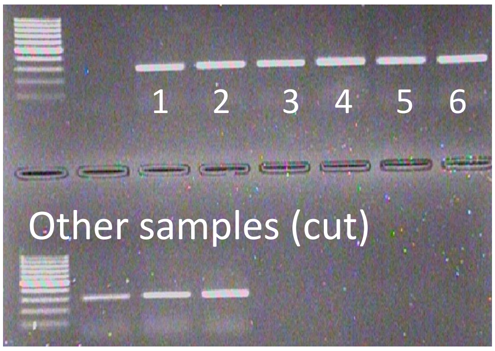

aas, 54.5 C, 28 cycles

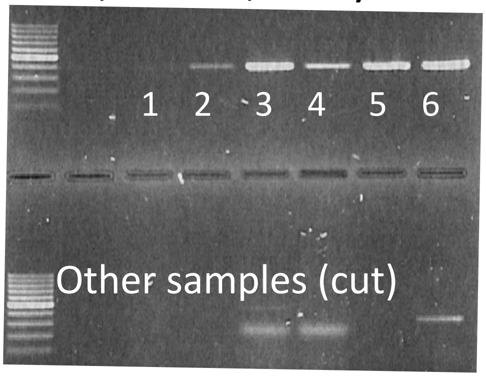

rbcL 58 C, 23 cycles

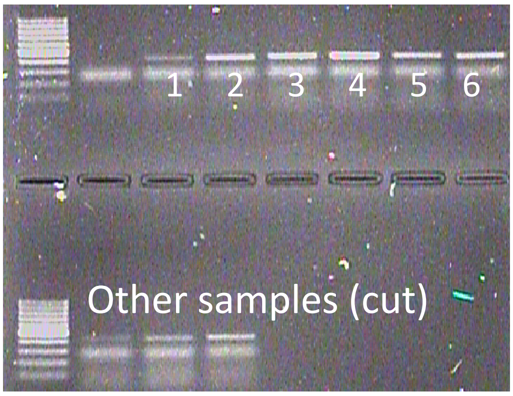

glpD, 53.5 C, 26 cycles

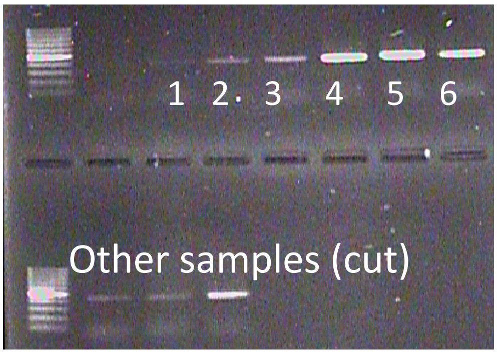

16S, 56 C, 13 cycles

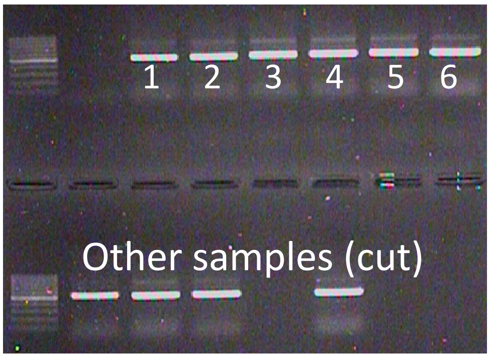

Supplement: Supplementary file 1 — Supplementary Data. [file 41598_2020_61100_MOESM1_ESM.pdf]
